# The associations of cardiovascular and lifestyle factors with mortality from chronic kidney disease as the underlying cause: the JACC study

**DOI:** 10.4178/epih.e2024077

**Published:** 2024-09-13

**Authors:** Shuai Guo, Tomoko Sankai, Kazumasa Yamagishi, Tomomi Kihara, Akiko Tamakoshi, Hiroyasu Iso, for the JACC Study Group

**Affiliations:** 1 Department of Public Health Medicine, Institute of Medicine, and Health Services Research and Development Center, University of Tsukuba, Tsukuba, Japan; 2 Doctoral Program in Public Health, Graduate School of Comprehensive Human Sciences, University of Tsukuba, Tsukuba, Japan; 3 Department of Public Health and Nursing, Institute of Medicine, University of Tsukuba, Tsukuba, Japan; 4 Department of Public Health, Graduate School of Medicine, Juntendo University, Tokyo, Japan; 5 Department of Public Health, Faculty of Medicine, Hokkaido University, Hokkaido, Japan; 6 National Center for Global Health and Medicine, Bureau of International Cooperation, Institute for Global Health Policy Research (iGHP), Tokyo, Japan

**Keywords:** Chronic kidney disease, Epidemiology, Risk factors

## Abstract

**OBJECTIVES:**

This study investigated conventional cardiovascular and lifestyle risk factors affecting mortality from chronic kidney disease as the underlying cause in the general Japanese population.

**METHODS:**

We conducted an 18.8-year follow-up study of 44,792 men and 61,522 women aged 40-79 from the Japan Collaborative Cohort Study for Evaluation of Cancer Risk between 1986 and 1990. Cox proportional hazard models were used to analyze the association between risk factors and mortality from chronic kidney disease.

**RESULTS:**

During the follow-up period, 373 participants (185 men and 188 women) died from chronic kidney disease. A body mass index of ≥27.0 kg/m^2^ (hazard ratio [HR], 2.00; 95% confidence interval [CI], 1.19 to 3.36 for men and HR, 1.91; 95% CI, 1.19 to 3.07 for women, compared with 23.0-24.9 kg/m^2^), a history of hypertension (HR, 2.32; 95% CI, 1.67 to 3.22 for men and HR, 2.01; 95% CI, 1.44 to 2.81 for women) and a history of diabetes mellitus (HR, 5.21; 95% CI, 3.68 to 7.37 for men and HR, 7.10; 95% CI, 4.93 to 10.24 for women) were associated with an increased risk of mortality from chronic kidney disease in both genders. In men, smoking was also associated with an increased risk (HR, 1.91; 95% CI, 1.25 to 2.90), while current drinking (HR, 0.58; 95% CI, 0.34 to 0.98 for <23 g/day; HR, 0.48; 95% CI, 0.29 to 0.80 for 23-45 g/day and HR, 0.53; 95% CI, 0.32 to 0.86 for ≥46 g/day) and exercising ≥5 hr/wk (HR, 0.42; 95% CI, 0.18 to 0.96) were associated with a lower risk. Similar but non-significant associations for smoking and drinking were observed in women.

**CONCLUSIONS:**

In addition to a history of hypertension and a history of diabetes mellitus, body mass index, smoking status, drinking status, and exercise habits were associated with the risk of mortality from chronic kidney disease.

## GRAPHICAL ABSTRACT

[Fig f1-epih-46-e2024077]

## Key Message

Hypertension and diabetes are the two leading causes of chronic kidney disease. In this study, we found that body mass index, smoking status, drinking status, and exercise habits, independent of hypertension and diabetes, were associated with mortality from chronic kidney disease. These findings underscore the importance of addressing these modifiable lifestyle factors.

## INTRODUCTION

At present, it is estimated that 10.2 million Japanese adults—or 10% of the adult population—have chronic kidney disease (CKD), which places them at a heightened risk of progression to end-stage renal disease (ESRD) [1,2]. Data from the United States Renal Data System indicate that in 2020, the prevalence of treated ESRD per million population in Japan was 2,749; this is the third-highest rate globally, following Taiwan and Korea [3]. Moreover, CKD is associated with an increased risk of cardiovascular disease and higher mortality rates, even in the early stages of the disease before the onset of dialysis [5].

Previous studies have shown that atherosclerosis and glomerulosclerosis share several pathobiological features, such as damage to endothelial cells, proliferation of vascular smooth muscle cells, and infiltration by monocytes and lymphocytes [6,7]. Therefore, factors that influence the onset and progression of cardiovascular disease may also affect the initiation and progression of CKD. Numerous epidemiological studies have identified risk factors for the development and progression of cardiovascular disease, including diabetes mellitus, hypertension, dyslipidemia, and several modifiable lifestyle factors such as higher body mass index (BMI) [8], smoking habits [9], drinking habits [10], and physical inactivity [11]. However, there are limited reports on the association between these modifiable lifestyle factors and the risk of CKD, and some findings have been contradictory. For example, a cohort study in the United States found that a higher BMI had a detrimental effect on renal function [12]. In contrast, another longitudinal study in the United States found that higher BMI was not associated with the incidence of CKD, although it was inversely associated with a composite outcome of CKD and all-cause mortality [13]. The relationship between alcohol intake and CKD has also shown inconsistent results, with studies reporting U-shaped, J-shaped, positive, or inverse associations [14-18]. A previous Japanese cohort study found an association between physical activity and the risk of CKD during 1 year of follow-up [19]. Given the limited and contradictory evidence, we utilized data from the Japan Collaborative Cohort Study for Evaluation of Cancer Risk (JACC study) to examine the associations of lifestyle factors, including BMI, drinking, smoking, physical activity, and cardiometabolic risk factors, with the risk of death from CKD in the general adult Japanese population.

## MATERIALS AND METHODS

### Study population

The sampling methods and protocols of the JACC study, sponsored by the Ministry of Education, Culture, Sports, Science and Technology, are detailed in other sources [20]. From 1986 to 1990, a total of 110,585 individuals—46,395 men and 64,190 women—ranging in age from 40 years to 79 years, across 45 areas in Japan, filled out self-administered questionnaires regarding their lifestyles and medical histories.

We excluded 4,271 participants with a history of renal disease from our analysis, leaving a cohort of 106,314 participants (44,792 men and 61,522 women) for follow-up in the present study.

### Mortality surveillance

Follow-up continued until the end of 2009, except for 10 areas (until 1999 in 4 areas, until 2003 in 4 areas, and until 2008 in 2 areas). Mortality surveillance data were entered at the public health center in the area of residency in each community, and then sent centrally to the Ministry of Health and Welfare. The underlying causes of death were coded for the National Vital Statistics according to International Classification of Diseases, 10th revision. Registration of death is required by the Family Registration Law in Japan. Therefore, all deaths that occurred in the cohort were ascertained by death certificates from a public health center. Individuals who moved out of the communities or died of other causes were treated as censored participants.

We defined the endpoint as death from CKD, using ICD-10 codes E10.2, E11.2, E12.2, E13.2, E14.2, I12-I13.9, N02-N08.8, N15.0, and N18-N18.9. This definition encompasses not only general CKD (N18-N18.9) but also CKD attributable to diabetes mellitus (E10.2, E11.2, E12.2, E13.2, E14.2), hypertension (I12-I13.9), glomerulonephritis (N03-N06.9), and other causes (N02-N02.9, N07-N08.8, N15.0) [21].

### Baseline survey

A self-administered questionnaire was used to collect baseline data, which included demographic characteristics (age, gender, height, and weight), lifestyle factors (smoking, drinking, physical activity, and dietary habits), and medical history of several diseases (hypertension, diabetes mellitus, and cancer). We investigated both traditional and non-traditional risk factors as potential predictors of mortality from CKD, including age, gender, BMI, smoking status, drinking status, weekly exercise hours, daily walking hours, and histories of hypertension and diabetes mellitus. BMI was calculated by dividing the self-reported weight in kilograms (participants were asked to report their adult weight) by the square of their self-reported height in meters. This calculation was used to categorize the population into 6 groups (< 19.0, 19.0-20.9, 21.0-22.9, 23.0-24.9, 25.0-26.9, and ≥ 27.0 kg/m^2^). Smoking status was categorized based on participants’ responses as never, former, or current smoking. Similarly, alcohol consumption status was categorized as never, former, or current drinking. The question regarding the amount of alcohol consumed per occasion referred to the Japanese drinking unit gou, which is equivalent to 23 g of ethanol. Current drinkers were further classified into 3 categories based on the amount of ethanol consumed per occasion: 1-22 g, 23-45 g, and ≥ 46 g.

### Statistical analysis

Statistical analyses focused on gender-specific mortality over the follow-up period from 1986 to 2009, which represents the longest duration of follow-up. For each participant, person-years were calculated from the start of the study until the date of death, relocation out of the community, or the end of the follow-up period, whichever came first.

Baseline characteristics are presented as mean± standard deviation (SD) for continuous variables and as percentages for categorical variables. The variables examined included age, BMI categories (< 19, 19-21, 21-23, 23-25, 25-27, and ≥ 27 kg/m^2^), exercise frequency (seldom or never, 1-2, 3-4, and ≥ 5 hr/wk), walking duration (seldom or never, 0.5, 0.6-0.9, and ≥ 1.0 hr/day), smoking status (never, former, and current smoking), drinking status (never, former, current drinking of 1-22 g, current drinking of 23- 45 g, and current drinking of 46 g or more ethanol per occasion), history of hypertension (yes/no), and history of diabetes mellitus (yes/no). All covariates were incorporated into the Cox proportional hazard models, which were stratified by gender. Missing values for the variables were addressed by creating additional categories labeled as “missing,” and dummy variables for these categories were included in the model.

Because renal function decline is associated with death from any cause [4], deaths due to other causes may preclude CKD death. Therefore, we also employed competing risk analysis using the Fine and Gray model to examine the association of all risk factors with CKD mortality. Deaths from all other causes were treated as competing risk events. Statistical analysis was conducted using SAS version 9.4 (SAS Institute Inc., Cary, NC, USA). A p-value less than 0.05 was considered statistically significant.

### Ethics statement

Individual informed consent before participation in the study was obtained in 36 of the 45 study areas, with written informed consent in 35 areas and oral consent in 1 area. In the remaining 9 areas, group consent was obtained from the respective area leaders. This study protocol including informed consent procedures was reviewed and approved by the Ethics Committee of Hokkaido University (approval No. I-14-044).

## RESULTS

Among the 106,314 participants (44,792 men and 61,522 women) followed for a median of 18.8 years, 373 individuals (185 men and 188 women) died from CKD. Of the men, 110 deaths were attributed to CKD (N18, N18.9), 61 to CKD due to diabetes mellitus, and 14 to CKD due to glomerulonephritis. Among the women, there were 116 deaths from CKD (N18, N18.9), 58 from CKD due to diabetes mellitus, 13 from CKD due to glomerulonephritis, and 1 from CKD due to other causes (Table 1). The mean age at entry for those who died from CKD was 64.5 years with an SD of 8.5 years for men and 67.2 years with an SD of 7.5 years for women.

Table 2 presents the baseline characteristics of the participants followed in the study. The mean values for age and BMI, along with the prevalence of a history of hypertension and diabetes mellitus, were higher among participants who died from CKD than among others, across both genders. Among women, the proportions of those who engaged in exercise for 5 or more hours per week and those who were former or current smokers were higher in those who died from CKD than in others. For men, the proportion of current smokers was also higher among those who died from CKD than among others.

Age-adjusted and multivariable gender-specific hazard ratios of CKD mortality for various exposure variables are shown in Table 3. In the age-adjusted model, a BMI ≥ 27.0 kg/m^2^, a history of hypertension, and a history of diabetes mellitus were associated with an increased risk of mortality from CKD in both genders. Current smoking was associated with an increased risk of mortality from CKD in men, while former smoking and former drinking were associated with an increased risk among women. In men, exercising ≥ 5 hr/wk, walking ≥ 1 hr/day, and consuming 23-45 g and ≥46 g of ethanol per occasion were associated with a decreased risk of mortality from CKD. Similar but non-significant associations with smoking status, drinking status, and hours of exercise and walking were observed in women.

The results from the multivariable model were consistent with those from the age-adjusted model, with a few exceptions. In men, the number of walking hours was no longer statistically significant. In women, neither former smoking nor former drinking remained statistically significant. However, in men, ethanol intake of less than 23 g per occasion was found to be statistically significant.

The associations between lifestyle factors, cardiometabolic risk factors, and CKD mortality remained consistent in both men and women when analyzed using competing risk analysis. The only exception was the lack of statistical significance for men who exercised for ≥ 5 hr/wk, which had a hazard ratio of 0.47 (95% CI, 0.21 to 1.07).

## DISCUSSION

In our current study, we found that a BMI of 27.0 kg/m^2^ or above, current smoking, a history of hypertension, and a history of diabetes mellitus were associated with a higher risk of death from CKD. Exercising for more than 5 hr/wk and current drinking were associated with a lower risk of death from CKD.

Some studies have found an association between BMI and CKD, while others have not observed such a relationship. For instance, in an American prospective study involving 11,104 healthy men, those in the highest BMI quintile (> 26.6 kg/m^2^) exhibited a higher risk of incident CKD than participants in the lowest quintile (< 22.7 kg/m^2^), with an odds ratio (OR) of 1.45 and a 95% CI of 1.19 to 1.76; the trend was statistically significant (p< 0.001) [12]. Similarly, a prospective study of 62,249 healthy, young, and middle-aged Korean individuals found that being overweight (BMI= 23.0-24.9 kg/m^2^) or obese (BMI > 25.0 kg/m^2^), even without metabolic abnormalities, was associated with a higher incidence of CKD [22]. However, other cohort and case-control studies did not demonstrate a significant association between BMI and CKD [13,23]. In Japan, while an association between the binary variable of obesity and CKD has been reported [14], no study has yet explored the relationship between individual BMI categories and CKD risk.

There is limited evidence regarding the impact of physical activity on CKD risk factors in healthy individuals. A cohort study involving 9,082 healthy adults from the National Health and Nutrition Examination Survey II in the United States found that physical inactivity was linked to an increased risk of ESRD and CKD mortality [24]. Conversely, another cohort study with 4,902 healthy adults in Japan found no significant relationship between physical activity and CKD risk, as defined by the onset of proteinuria [19]. In our study, we observed a significant inverse relationship between hours of exercise and CKD mortality, but only among men. Therefore, our findings, along with those from previous studies, are inconsistent, indicating that further research is needed to clarify the effects of exercise on CKD in healthy populations.

The relationship between alcohol consumption and the development of CKD has been a subject of debate in the literature. A prospective cohort study involving 1,658 women nurses in the United States found that light to moderate alcohol intake did not adversely affect renal function [25]. Conversely, a cohort study with 3,392 American participants indicated that heavy drinking, defined as consuming 4 or more servings of alcohol per day, was associated with an increased risk of CKD [15]. Studies from Japan and the Netherlands reported an inverse association between alcohol consumption and the risk of CKD [14,18]. In our study, we observed an inverse association between alcohol consumption and mortality from CKD, even though the alcohol intake exceeded the recommended levels for the Japanese population (less than 20 g/day). The biological mechanisms by which alcohol consumption may reduce the risk of CKD remain unclear. Clinical trials have demonstrated that light to moderate alcohol consumption can increase serum high-density lipoprotein cholesterol [18,26], decrease plasma fibrinogen levels [26], and inhibit induced platelet aggregation [26]. Additionally, autopsy data from the Honolulu Heart Program revealed a strong protective association between alcohol intake and hyalinization of renal arterioles [27]. Arteriosclerotic changes in the kidneys are a major cause of the decline in the estimated glomerular filtration rate (GFR) [28].

Several studies have shown associations between smoking and both the onset of CKD and progression to ESRD [14,29-33]. A cross-sectional study involving 7,476 participants in the Netherlands revealed a dose-dependent relationship between the number of cigarettes smoked and several renal indicators, including microalbuminuria, albuminuria, elevated GFR, and decreased GFR [33]. Similarly, a prospective cohort study of 65,589 participants in Norway found a dose-dependent association between the number of cigarettes smoked and the risk of kidney failure. Additionally, the study observed a dose-dependent relationship between smoking cessation and a reduced risk of kidney failure in men [32]. In terms of the mechanisms by which smoking contributes to kidney dysfunction, several have been proposed. These include endothelial cell dysfunction, the formation of advanced glycation end products, and insulin resistance [34]. These factors may create a hypoxic and ischemic environment in the renal arterial system, leading to morphological changes such as glomerular sclerosis, tubular atrophy, and interstitial fibrosis. Ultimately, these changes contribute to a decline in renal function [35].

Our study found that a history of hypertension and diabetes mellitus were associated with higher CKD mortality, aligning with previous research [31,36-40]. A retrospective cohort study from a hypertension disease registry in the Denver metropolitan area, United States, revealed that each 10-mmHg increase in baseline systolic blood pressure corresponded to a 6% heightened risk of developing CKD [37]. Similarly, a prospective cohort study in China demonstrated a strong, graded relationship between blood pressure and the risk of ESRD [38]. Regarding diabetes mellitus, a cohort study involving 1,574,749 participants from England and Wales identified a 6-fold increase in the risk of moderate to severe CKD associated with the condition [40]. Another study, which included 23,534 Americans, found a 5-fold increase in risk for men and an 11-fold increase for women for ESRD and CKD death associated with diabetes mellitus [31].

This study has several limitations. First, we only had access to data on cardiovascular and lifestyle factors at baseline, which prevented us from accounting for changes in these factors over time. Second, we lacked baseline data on serum creatinine values and proteinuria, which are essential for assessing renal function; we also excluded only a subset of participants who reported a history of renal disease. Third, our reliance on self-reported data for hypertension and diabetes could lead to under-reporting. Such under-reporting in cohort studies may introduce bias, potentially skewing the association towards null. Finally, CKD outcomes were determined based on the underlying cause of death listed on death certificates. Using CKD mortality as an outcome could result in the under-coding of early-stage CKD, failing to capture the full spectrum of the disease. Moreover, according to the rules governing death certificates, CKD patients who died from other causes, such as cardiovascular disease, were not classified as having CKD as the underlying cause of death. It is important to note that our outcomes are unlikely to include CKD with other underlying causes.

The strengths of our study include conducting a large cohort study within the Japanese general population. To our knowledge, this is the first report that examines the association between conventional cardiovascular and lifestyle risk factors and mortality from CKD.

In conclusion, both current smoking and a history of hypertension or diabetes mellitus were positively associated with the risk of CKD mortality. Conversely, current drinking and hours of exercise were inversely associated with this risk. Our findings suggest that modifying these risk factors could help prevent CKD-related deaths.

## Figures and Tables

**Figure f1-epih-46-e2024077:**
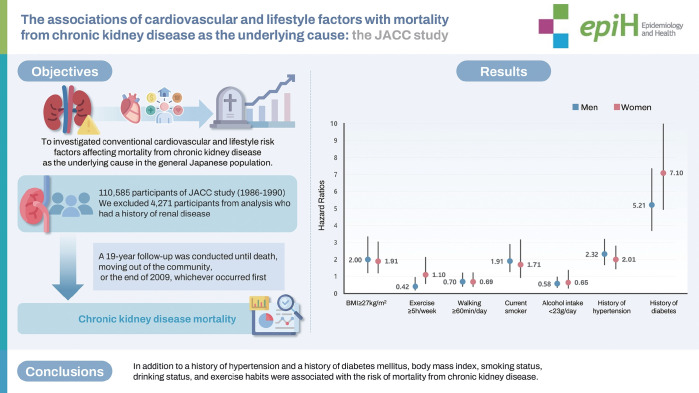


**Table 1.** Gender-specific causes of death due to CKD

Cause of death	ICD-10 code	Men (n)	Women (n)	Total (n)
CKD	N18, N18.9	110	116	226
CKD due to diabetes	E14.2	61	58	119
CKD due to glomerulonephritis	N03.9, N04.9, N05.9	14	13	27
CKD due to other causes	N02.8	0	1	1
Total	-	185	188	373

CKD, chronic kidney disease; ICD-10, International Classification of Diseases, 10th revision.

**Table 2.** Gender-specific characteristics of study participants

Characteristics	Men			Women		
	Total	Death from CKD	Others	Total	Death from CKD	Others
Age (yr)	57.6±10.2	64.5±8.5	57.5±10.2	57.8±10.1	67.2±7.5	57.8±10.1
BMI (kg/m^2^)	22.6±2.8	22.8±3.3	22.6±2.8	22.9±3.1	23.4±3.7	22.9±3.1
Hours of exercise (hr/wk)						
Seldom or never	54.8	48.4	54.8	60.1	49.5	60.2
1-2	13.5	13.5	13.5	10.9	9.0	10.9
3-4	5.8	7.6	5.8	4.3	4.8	4.3
≥5	5.9	3.2	5.9	3.7	5.3	3.7
Hours of walking (hr/day)						
Seldom or never	9.5	9.2	9.5	8.1	8.0	8.1
<0.5	14.6	15.1	14.1	13.2	12.8	13.2
0.5-1.0	14.7	14.6	14.7	15.5	11.2	15.5
≥1.0	37.6	29.2	37.6	38.6	29.3	38.6
Smoking status						
Never	19.5	15.7	19.5	80.1	70.7	80.1
Former	24.9	23.2	24.9	1.5	3.7	1.5
Current	50.7	55.1	50.7	4.8	5.9	4.8
Ethanol consumption (g/day)						
Never	17.9	25.4	17.9	66.8	67.6	66.8
Former	5.9	8.1	5.9	1.5	3.7	1.5
Current						
<23	13.8	10.8	13.8	9.5	3.7	9.5
23-45	18.3	12.4	18.3	1.6	0.5	1.6
≥46	23.3	14.1	23.3	0.5	0	0.5
History of hypertension	19.0	39.5	18.9	20.7	46.3	20.7
History of diabetes mellitus	6.0	27.6	5.9	3.6	24.5	3.5

Values are presented as mean±standard deviation or %. CKD, chronic kidney disease; BMI, body mass index.

**Table 3.** Gender-specific for chronic kidney disease according to lifestyle and cardiovascular risk factors^1^

Variables	Men					Women				
	No. at risk	No. of deaths	Person-years	Model 1	Model 2	No. at risk	No. of deaths	Person-years	Model 1	Model 2
Body mass index (kg/m^2^)										
<19.0	3,390	21	45,479	1.40 (0.83, 2.34)	1.52 (0.90, 2.57)	5,105	14	77,394	0.98 (0.54, 1.78)	1.21 (0.66, 2.21)
19.0-20.9	8,888	30	136,512	0.83 (0.53, 1.31)	0.87 (0.55, 1.38)	10,607	29	174,613	1.15 (0.72, 1.83)	1.38 (0.86, 2.20)
21.0-22.9	12,071	49	192,671	1.05 (0.71, 1.57)	1.05 (0.71, 1.57)	15,447	45	259,268	1.30 (0.86, 1.96)	1.36 (0.90, 2.06)
23.0-24.9	10,272	38	167,400	1.00 (reference)	1.00 (reference)	13,277	26	222,836	1.00 (reference)	1.00 (reference)
25.0-26.9	5,091	16	83,583	0.91 (0.52, 1.61)	0.83 (0.47, 1.47)	7,608	25	127,318	1.46 (0.89, 2.37)	1.37 (0.84, 2.24)
≥27.0	2,686	21	44,164	2.39 (1.43, 3.99)	2.00 (1.19, 3.36)	5,451	29	90,803	2.37 (1.48, 3.77)	1.91 (1.19, 3.07)
Hours of exercise (hr/wk)										
Seldom or never	24,540	89	388,347	1.00 (reference)	1.00 (reference)	37,000	93	606,948	1.00 (reference)	1.00 (reference)
1-2	6,044	25	95,914	1.10 (0.70, 1.71)	1.08 (0.69, 1.70)	6,698	17	109,423	0.92 (0.55, 1.55)	0.92 (0.55, 1.56)
3-4	2,584	14	39,088	1.15 (0.65, 2.03)	1.10 (0.62, 1.96)	2,645	9	42,716	0.95 (0.48, 1.88)	0.86 (0.43, 1.71)
≥5	2,625	6	39,294	0.39 (0.17, 0.89)	0.42 (0.18, 0.96)	2,266	10	35,897	1.00 (0.52, 1.92)	1.10 (0.57, 2.15)
Hours of walking (hr/day)										
Seldom or never	4,260	17	64,564	1.00 (reference)	1.00 (reference)	4,998	15	79,966	1.00 (reference)	1.00 (reference)
<0.5	6,297	28	96,083	0.94 (0.51, 1.71)	0.90 (0.49, 1.67)	8,110	24	126,067	0.83 (0.43, 1.57)	0.84 (0.44, 1.62)
0.5-1.0	6,586	27	101,516	0.79 (0.43, 1.44)	0.86 (0.46, 1.59)	9,505	21	151,645	0.61 (0.31, 1.18)	0.63 (0.32, 1.24)
≥1.0	16,828	54	269,944	0.58 (0.34, 1.00)	0.70 (0.40, 1.22)	23,738	55	394,065	0.62 (0.35, 1.09)	0.69 (0.39, 1.24)
Smoking status										
Never	8,731	29	143,741	1.00 (reference)	1.00 (reference)	49,289	133	822,961	1.00 (reference)	1.00 (reference)
Former	11,144	43	169,889	1.06 (0.66, 1.70)	1.04 (0.65, 1.67)	901	7	13,186	2.44 (1.14, 5.21)	1.78 (0.82, 3.87)
Current	22,721	102	357,950	1.72 (1.14, 2.61)	1.91 (1.25, 2.90)	2,933	11	45,832	1.73 (0.94, 3.20)	1.71 (0.92, 3.19)
Ethanol consumption (g/day)										
Never	8,028	47	121,493	1.00 (reference)	1.00 (reference)	41,097	127	676,508	1.00 (reference)	1.00 (reference)
Former	2,649	15	33,798	1.01 (0.56, 1.80)	0.81 (0.45, 1.46)	919	7	13,574	2.88 (1.35, 6.16)	1.98 (0.92, 4.30)
Current										
<23	6,162	20	100,473	0.63 (0.38, 1.07)	0.58 (0.34, 0.98)	5,816	7	95,188	0.59 (0.28, 1.27)	0.65 (0.30, 1.39)
23-45	8,177	23	129,321	0.54 (0.33, 0.90)	0.48 (0.29, 0.80)	1,002	1	15,899	-	-
≥46	10,414	26	166,562	0.61 (0.38, 0.99)	0.53 (0.32, 0.86)	321	0	5,061	-	-
History of hypertension										
No	31,568	92	516,294	1.00 (reference)	1.00 (reference)	42,192	75	718,527	1.00 (reference)	1.00 (reference)
Yes	8,492	73	119,654	2.44 (1.79, 3.34)	2.32 (1.67, 3.22)	12,754	87	195,030	2.58 (1.88, 3.53)	2.01 (1.44, 2.81)
History of diabetes mellitus										
No	36,445	113	586,737	1.00 (reference)	1.00 (reference)	51,079	109	859,672	1.00 (reference)	1.00 (reference)
Yes	2,701	51	36,935	6.14 (4.41, 8.56)	5.21 (3.68, 7.37)	2,216	46	29,668	8.84 (6.24, 12.5)	7.10 (4.93, 10.24)

Values are presented as hazard ratio (95% confidence interval).

1 Model 1: adjusted for age; Model 2: adjusted further for body mass index, hours of exercise, hours of walking, smoking status, drinking status, history of hypertension and history of diabetes mellitus, except for each exposure variable.
